# Evidence for lasting alterations to aquatic food webs with short-duration reservoir draining

**DOI:** 10.1371/journal.pone.0211870

**Published:** 2019-02-07

**Authors:** Christina A. Murphy, Ivan Arismendi, Gregory A. Taylor, Sherri L. Johnson

**Affiliations:** 1 Department of Fisheries and Wildlife, Oregon State University, Corvallis, Oregon, United States of America; 2 Willamette Valley Project, US Army Corps of Engineers, Lowell, Oregon, United States of America; 3 Pacific Northwest Research Station, US Forest Service, Corvallis, Oregon, United States of America; Southwest University, CHINA

## Abstract

Large dams and their respective reservoirs provide renewable energy and water security, but also can profoundly alter riverine ecosystems. Here, we present evidence of changing aquatic food web structure in the seasons following short-duration, extreme manipulation of water levels in a reservoir (i.e., draining of the reservoir to the original riverbed during fall to assist outmigration of juvenile Chinook Salmon). We find unintended and lagged consequences of transitioning from a lake to a river, even temporarily, that resulted in trophic shifts away from piscivory and towards feeding at lower trophic levels for two common piscivorous fishes in reservoirs. Using natural abundances of nitrogen stable isotopes, we observed lower trophic level of feeding for invasive Largemouth Bass (*Micropterus salmoides*) and native Rainbow Trout (*Oncorhynchus mykiss)* during the summers following reservoir refilling than in nearby reference reservoirs that were not temporarily drained during fall. Declines in trophic levels of aquatic top predators have been rarely documented outside of controlled laboratory conditions. While useful for assisting outmigration of juvenile salmonids, the temporary draining of a reservoir to riverbed can also result in novel shifts in foodweb dynamics including reduced piscivory. As large dams continue to be operated and constructed worldwide, increased understanding of the community and ecosystem-level effects of reservoir management will be critical to evaluating trade-offs between human water needs, conservation of high value species, and ecosystem services impacted by river fragmentation.

## Introduction

Worldwide over 58,000 large dams (> 15 m height) collectively store more than 6,000 km^3^ of freshwater [1–4]. Because these large dams and reservoirs frequently provide services including renewable energy and water security [5], many of them continue to be constructed worldwide [6]. Large dams and their respective reservoirs can profoundly alter riverine ecosystems including modification of hydrological regimes [7,8], degradation of habitat, loss of longitudinal connectivity [9,10], and proliferation of invasive species [11,12]. The artificial reservoir habitats created by dams [13] often contain a complex mixture of native and invasive species [14] and novel food webs [11]. Large dam operation must necessarily balance trade-offs between multiple objectives [15]. To this end, studies on new management strategies and manipulations of reservoir water levels may provide insight into trade-offs between ecological processes, ecosystem dynamics and water level management [16].

In the Pacific Northwest of North America (PNW), the river networks originally supported cold-water species, including anadromous salmonids. However, the large reservoirs inserted into these networks have created novel habitats that are now typically inhabited by both cold-water natives and warm-water invasive fishes [14]. Salmonids spawn in the headwaters of the river networks, upstream of the reservoirs, and upon hatching, the juvenile salmonids move downstream. During this downstream movement they frequently become entrained in the reservoirs that may represent ecological traps for outmigration of threatened and endangered salmonids [17]. Because entrained juveniles use the large reservoirs as rearing habitats, and those habitats contain large numbers of invasive piscivores, reservoirs put juvenile salmonids at increased risk of being prey for invasive warm-water fishes [18].

**Fig 1:**
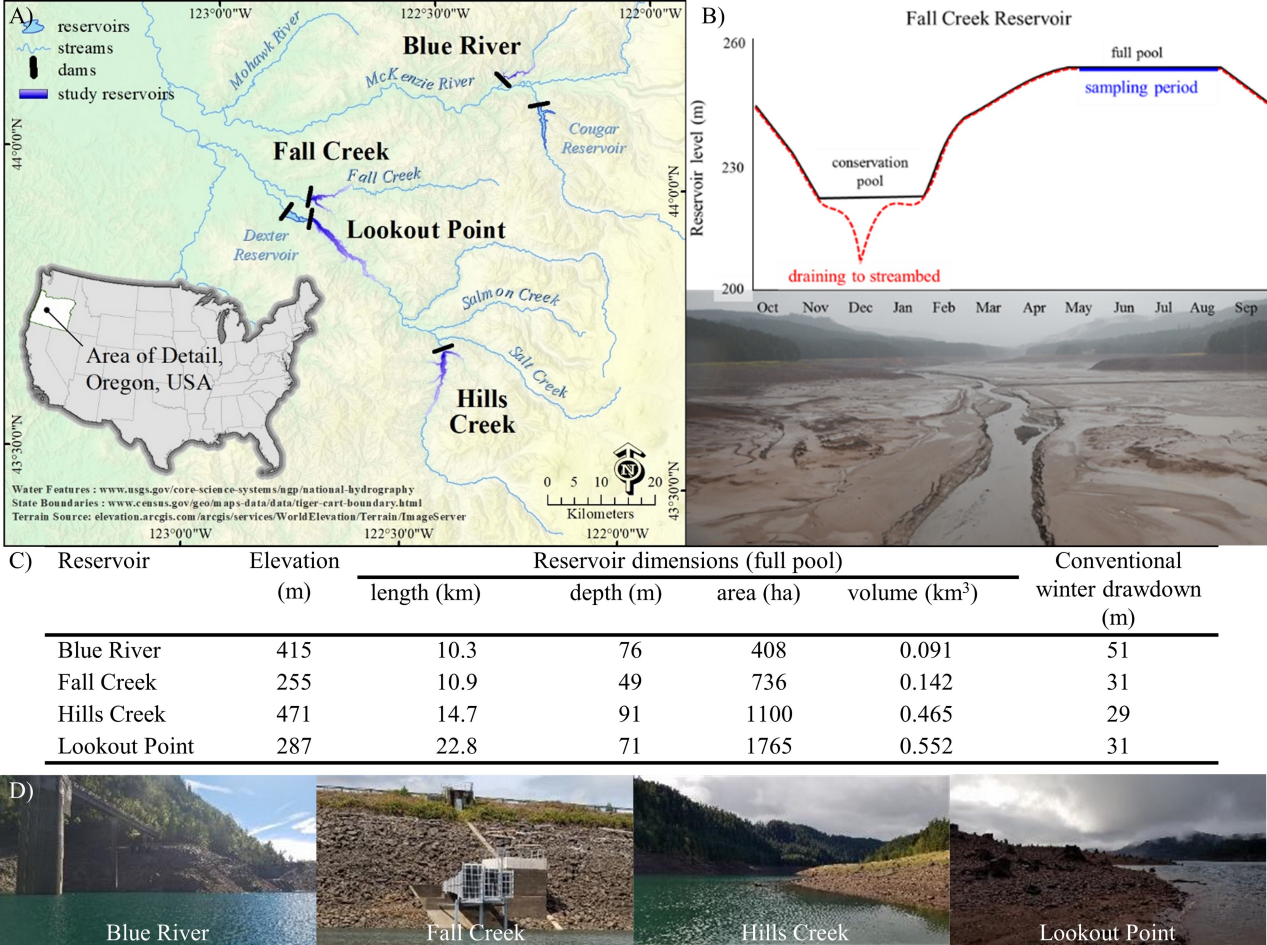
Study sites and management characteristics. (A) Map of the four Willamette Basin study reservoirs (purple). (B) The water control diagram with the conventional drawdown to conservation pool water level management (rule curve; black, solid) and the draining to riverbed (red, dashed) for the Fall Creek treatment reservoir. (C) Table of the conventional management characteristics of the study reservoirs. (D) Photographs from each reservoir in September, to illustrate exposed littoral area, when reservoirs are still accessible but water levels have begun to lower per conventional management. Photographs: C.A. Murphy. Fall Creek Reservoir is managed by short duration winter draining to riverbed (lowered by 49 m instead of the conventional 31 m) while the other reservoirs undergo conventional winter water level management (ranging from 29 m to 51 m in magnitude) and are reduced in lake levels but maintain a ‘minimum conservation pool’ (i.e. a small lake above riverbed). In conventional reservoir management each reservoir follows ‘rule curve’ diagrams for reservoir water levels to maintain downstream flow targets. During the winter, these large reservoirs are held at a reduced lake level, the ‘conservation pool’ that allows for the capture of peak inflows, reducing downstream flood risks. This period exposes littoral sediments. Captured water is used to fill the reservoir beginning late until ‘full pool’ is reached in spring. Stored water is then available to augment summer downstream flows as well as provide recreational opportunities in the reservoirs before the reservoir water level is reduced in the fall to the minimum lake level. The typical conservation pool is represented by a flat line during winter months and full pool by a flat line during summer months. Water control diagrams for all study reservoirs are available at: http://www.nwd-wc.usace.army.mil/nwp/teacup/willamette/.

To test how better to facilitate the downstream movement of juvenile salmon out of reservoirs, a new management strategy involving short duration draining of a reservoir to riverbed in late fall has been implemented. Rather than drawdown to an overwinter 'conservation' pool (to provide storage for winter rains and prevent downstream flooding), all standing water is now briefly drained in late fall to expose the riverbed. This flushes downstream most migratory salmonids along with other fishes. This week-long event is followed by refill to the traditional winter conservation pool and conventional spring and summer water level management (Fig 1B).

We hypothesized that draining of the reservoir might alter food web structure upon refilling, due to changes in lower trophic resources. We expected that decreased densities of zooplankton, and increased concentrations of nutrients and higher turbidity from resuspension of sediments, would lead to increased predation on fishes by piscivores. Collectively, changes in reservoir ecology could result from management actions to improve fish passage. We aimed to evaluate unexpected consequences at the whole food web level to aid in the future transferability of lessons learned here.

## Results

**Fig 2:**
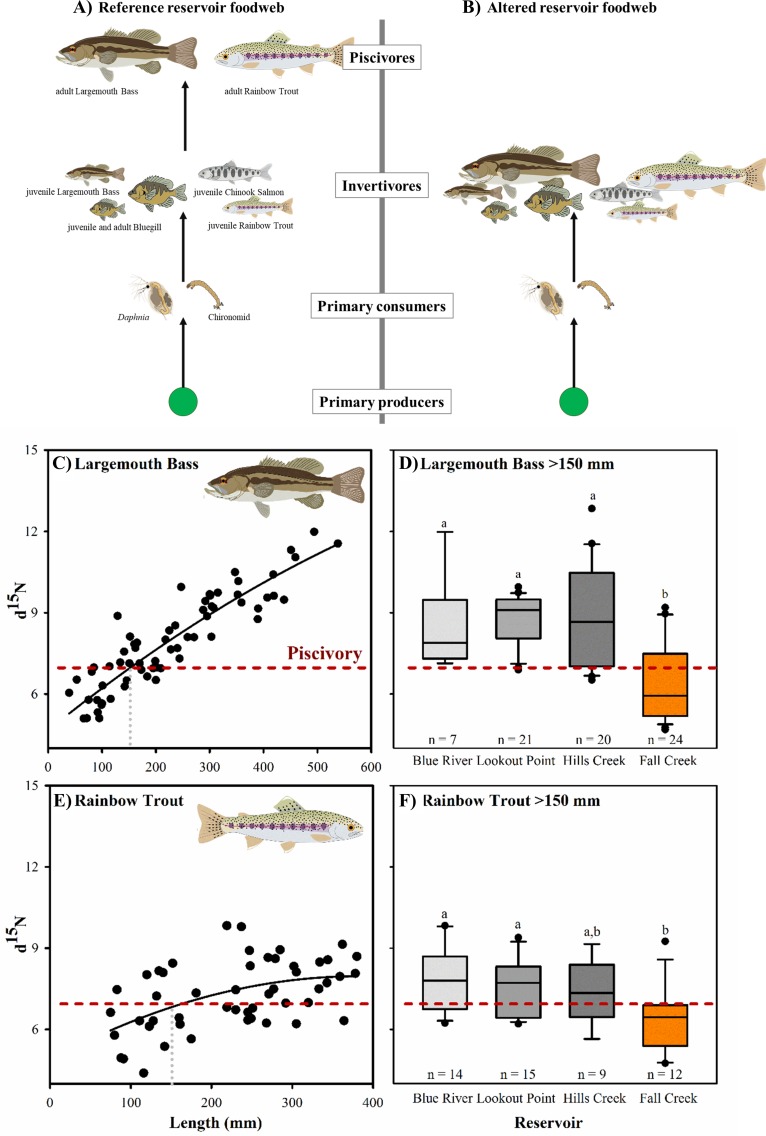
**Trophic levels of key taxa (top) and δ^15^N ratios for Largemouth Bass and Rainbow Trout (bottom).** (A) Reference reservoir food chain compared to the (B) altered reservoir food chain observed in the previously drained reservoir. Additional species found in Willamette Basin reservoirs but not pictured here include: bullhead catfish, Largescale Sucker and sculpin (detritivores); dace, mosquitofish, Redside Shiner, and Threespine Stickleback (invertivores); Bull Trout, crappie, Northern Pikeminnow, Walleye and Yellow Perch (juvenile invertivores, adult piscivores). (C and D) δ^15^N ratios increase naturally with increasing size of fish in reference reservoirs. The threshold for piscivory is indicated by a red dashed line. The grey dotted vertical line indicates the 150 mm threshold, above which fish diets are predominantly piscivorous. Although both species are capable of consuming fish as prey when smaller than 150 mm, this size threshold was calculated as the intersection of these (C and D) reference ontogenetic trajectories and the predicted piscivory isotopic ratio. (E and F) δ^15^N values of large (>150 mm) (E) Largemouth Bass and (F) Rainbow Trout in Fall Creek Reservoir (shown in orange) are lower than in other reservoirs (shown in gray; S1 Table). Fall Creek experienced riverbed draining while other reservoirs maintained conservation pools. Lower δ^15^N values indicate that consumers are feeding lower on the food chain.

After refilling of a large reservoir that has been temporarily drained to its original riverbed the previous fall, we observed that nitrogen stable isotope ratios (δ^15^N) of large, normally piscivorous fish—Largemouth Bass (*Micropterus salmoides*) and Rainbow Trout (*Oncorhynchus mykiss)* larger than 150 mm in fork length—were lower in Fall Creek Reservoir than in nearby reference reservoirs. This suggests that these top aquatic predators were no longer feeding on fish. Likely, they switched diets toward consuming invertebrates at lower trophic levels (Fig 2). Our findings are based on sampling in four large reservoirs (400–1,700 ha) during summer full pool. We compared the manipulated reservoir (Fall Creek) with three nearby reservoirs in the Willamette Valley, Oregon, USA (Fig 1). Because these reservoirs are managed for multiple purposes, all of them have substantial water level fluctuations throughout the year, and are drawn down in fall to a small conservation pool so that winter rains can be stored and large floods avoided (Fig 1). To increase downstream passage of salmonids in late fall, Fall Creek Reservoir is further drained and riverbed exposed for approximately one week.

To validate that the lower δ^15^N in these two predatory fish were not due to differences in basal resources among reservoirs, we compared nitrogen stable isotopes ratios from multiple tropic levels across reservoirs. Stable isotope ratios from lower trophic levels were consistent among reservoirs (Table 1). Mean phytoplankton δ^15^N values were less than 1‰ at all reservoirs. Zooplankton and chironomids were less than 2.5‰ whereas fish values averaged 4–6‰ for lower trophic levels (e.g. Bluegill) and more than 7‰ for piscivorous fishes (e.g. large Largemouth Bass and Rainbow Trout). Average ratios for detritivorous fishes in Fall Creek (e.g. bullhead) were intermediate to the other reservoirs where they were detected.

**Table 1:** δ^15^N values for other trophic levels and fish species in the four study reservoirs. Note that phyto- and zoo-plankton samples were bulk samples and likely represent a range of taxa.

δ^15^N	Blue River Reservoir	Fall Creek Reservoir	Hills Creek Reservoir	Lookout Point Reservoir
**Phytoplankton**	0.44 (n = 2)	**0.77 (n = 6)**	0.74 (n = 5)	0.29 (n = 4)
**Zooplankton**	0.01 (n = 3)	**1.46 (n = 3)**	1.17 (n = 3)	*
**Chironomidae (aquatic macroinvertebrate)**	0.28 (n = 1)	**2.05 (n = 3)**	2.15 (n = 4)	0.73 (n = 1)
**Bluegill (*Lepomis macrochirus*)**	4.27 (n = 9)	**4.97 (n = 12)**	5.76 (n = 10)	5.16 (n = 8)
**Brown Bullhead (*Ameiurus nebulosus*)**	Not detected	**6.58 (n = 6)**	7.69 (n = 5)	6.27 (n = 12)
**Chinook Salmon (*Oncorhynchus tshawytscha*)**	Not present	**6.08 (n = 49)**	7.23 (n = 71)	7.97 (n = 29)
**Largemouth Bass (*Micropterus salmoides*) < 150 mm**	5.29 (n = 5)	**5.19 (n = 15)**	6.16 (n = 11)	6.68 (n = 6)
**Largemouth Bass (*Micropterus salmoides*) > 150 mm**	8.60 (n = 7)	**6.61 (n = 18)**	8.94 (n = 20)	8.77 (n = 20)
**Rainbow Trout (*Oncorhynchus mykiss*) < 150 mm**	4.88 (n = 2)	**5.12 (n = 1)**	6.57 (n = 9)	6.94 (n = 3)
**Rainbow Trout (*Oncorhynchus mykiss*) > 150 mm**	7.88 (n = 14)	**6.36 (n = 12)**	7.42 (n = 9)	7.56 (n = 14)
**Yellow Bullhead (*Ameiurus natalis*)**	Not detected	**6.64 (n = 18)**	7.19 (n = 4)	6.97 (n = 10)

Mean δ^15^N values and sample sizes (n) for other trophic levels and fish species in the four study reservoirs. Data represent either whole organisms (plankton and macroinvertebrates) or individual fin clips (fishes). The riverbed draining treatment reservoir is highlighted in bold.

*Bulk zooplankton were not collected for Lookout Point but the mean δ^15^N for Daphniidae was 1.56 (n = 7).

The turnover time for fish fin tissue allowed us to be confident that observed changes in isotopic ratios were not due to differences in diet during the short duration draining to riverbed in the previous fall [19]; observed isotopic ratios most likely represented diets during the current summer full pool. Statistically significant differences in δ^15^N observed for common predatory fish at Fall Creek Reservoir could be explained by a greater contribution of lower trophic level prey (i.e. invertebrates) in their diet. We also noted lower content of prey fish in the stomachs of captured Largemouth Bass in Fall Creek Reservoir than in other reservoirs (S2 Table); however, the number of fish captured and examined were limited.

## Discussion

Our findings illustrate that an unconventional short-duration draining of a reservoir to its original riverbed may have important unexpected consequences on the following season's food webs. The predatory fish that remained in the reservoir after draining and refilling occupied a different trophic position than the same species in other reservoirs. Instead of keep preying upon other fishes, they appeared to have consumed less isotopically enriched prey, likely invertebrates and zooplankton.

Within the literature, rarely has it been documented that fish species shift from piscivory to the consumption of lower trophic levels; such changes are infrequently noted due to seasonal prey availability [20,21] and in small-scale controlled experiments [22]. To our knowledge, one of the only large scale examples of this shift has been observed following introduction of competitor species; invasive fishes have been shown to cause previously dominant species to shift their diet to lower quality food from lower trophic levels due to competition for high quality food [23]. During development, the direction of diet switching in fishes is typically on the opposite side from consumption of lower trophic levels towards higher trophic levels [24]. This is due to larger body sizes individuals require more energy [25]. Larger fishes optimize their energy budget by simultaneously minimizing outgoing energy costs of foraging [26] while choosing prey items with the highest energy content possible, generally from higher trophic levels [27].

### Possible mechanisms for observed isotopic ratios of predators in Fall Creek

**Fig 3:**
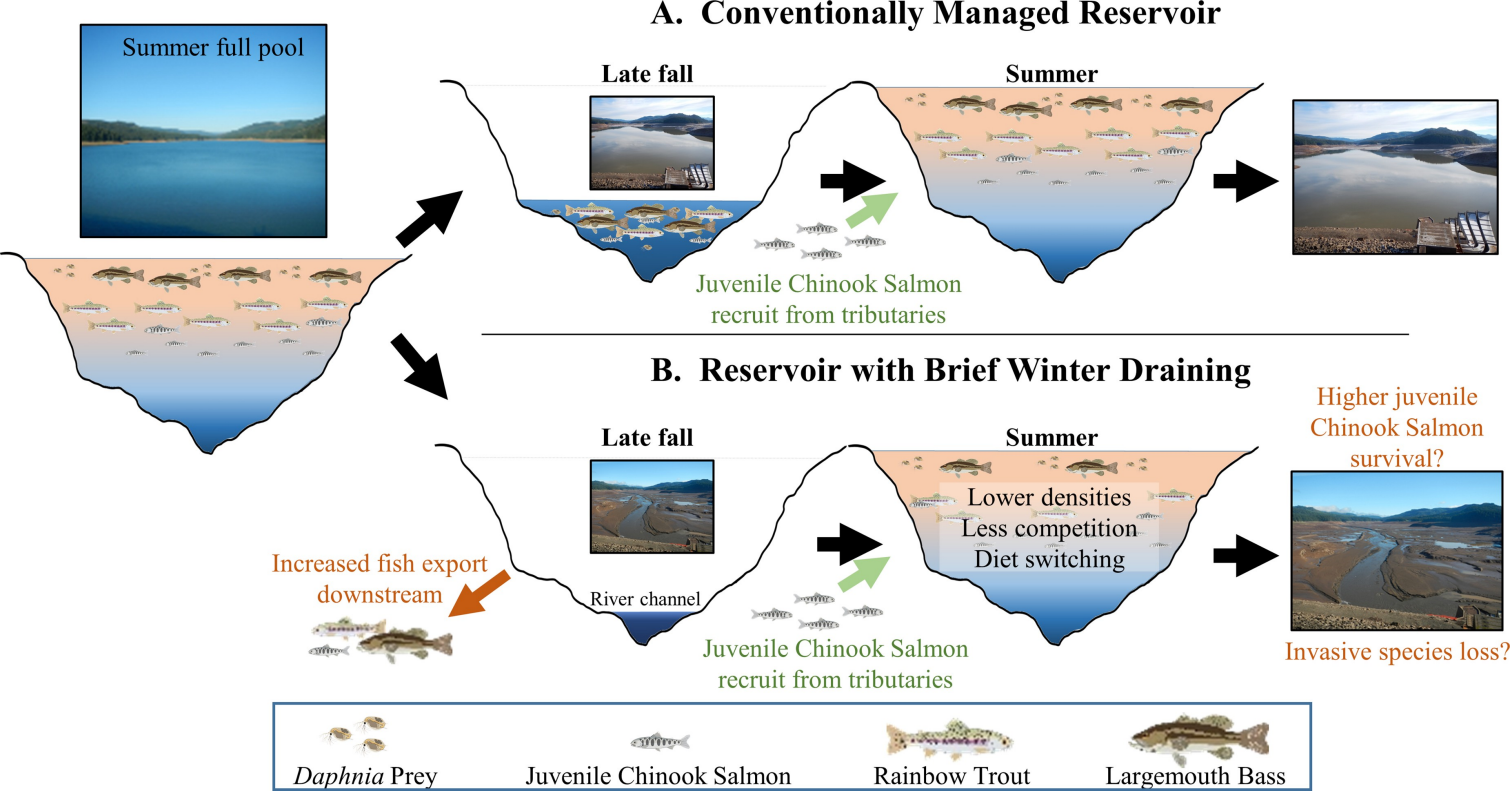
Hypothesized progression of the ecosystem-level water management cycle in reservoirs and ecological processes. A full pool reservoir during summer is followed by (A) conventional lowering to minimum reservoir level or (B) experimental draining of the reservoir to riverbed during late fall, and refilling in spring. Under both scenarios, much of the benthos are exposed. In the case of Fall Creek Reservoir, this draining results in lotic conditions, with no holding pool, for one week annually. Sediments do not appear to desiccate during this period. The draining of a reservoir to riverbed assists anadromous Chinook Salmon with downstream migration, but acts as a pulse disturbance washing out many reservoir resident fishes. Upon refill, the reservoir in (B) has reduced densities of overwintering fish species (zooplankton and benthic invertebrates can quickly recover to previous densities from egg banks and resting stages). This results in shifts in the trophic dynamics for predatory fish, presumably through the reduction of small prey fishes. Though juvenile Chinook Salmon hatch in streams and move to the reservoirs in early spring, their densities are relatively low, making a piscivorous strategy which relies on them alone inefficient and thus, predaceous fishes now focus on invertebrate prey instead.

There are several possible mechanisms for the lower δ^15^N of large Largemouth Bass and Rainbow Trout after draining and refilling of Fall Creek Reservoir. The most likely is that a change in the densities of prey fishes was responsible for altering the feeding by these piscivores and food web relationships [23]. Many small fishes, both salmonids and others, were probably flushed downstream during the fall draining and after refilling there could be a reduction in availability of high quality prey. This conceptual model of reduced densities of prey fish following refilling (Fig 3) is supported by decreased numbers of invasive species exported from Fall Creek Reservoir relative to annual juvenile Chinook Salmon outmigrants in the subsequent years [28]. At a reference reservoir, there have not been similar changes in numbers of prey fish leaving the reservoir.

We do not find evidence that these piscivores increased their consumption of juvenile Chinook Salmon, who enter the reservoir each spring after hatching upstream. If juvenile salmon had been an increasing part of a predominantly piscivorous diet, the δ^15^N signals/values of piscivores should have increased. Predation on these threatened and endangered juvenile salmon by native and invasive fish in reservoirs is a concern of managers [17], and the isotopic ratios observed in the previously drained reservoir suggest predation pressures may be reduced with this management.

The similarity of isotopic ratios of basal resources among reservoirs as well as densities of zooplankton and invertebrates suggest that observed isotopic differences for Largemouth Bass and Rainbow Trout are not associated with differences in those resources (Table 1 and S3 Table). The decrease in nitrogen isotopic enrichment is also not consistent with lack of food or starvation of these piscivorous species. In a catabolic state, we would expect isotope levels to become more enriched and for starving fish to then appear to be more, not less, piscivorous [29–31]. Reductions in visibility could also alter foraging success, though previous research suggests that piscivory in both Largemouth Bass and Rainbow Trout increases with increasing turbidity [32,33]. Therefore, it would not seem that differences in light transmission at depth, a function of increased resuspension of fine sediment, would explain the reduced piscivory observed in the drained reservoir (S4 Table).

It is also possible that declines in fish recruitment success, although delayed, could happen as a consequence of stress from the short duration draining to riverbed. This would result in a reduction in the density of prey fishes, including young of both piscivorous and non-piscivorous species. Regardless, without abundant prey fishes, top fish predators capable of omnivory may alter their feeding behavior to prioritize lower trophic level prey, as the behaviors related to search and capture are prey-dependent [34,35].

### Tradeoffs

The timing and duration of the draining of a reservoir in late fall appears to result in minimal impacts to the social and economic benefits of the dam operation; winter flood control capacity is still maximized and spring and summer recreation are not impacted. Modification of reservoir operations may provide a tool to support salmon recovery in western North America by providing downstream passage and altering the foraging of invasive piscivores in a way that could reduce predation on juvenile salmon, without depleting the *Daphnia* prey commonly consumed by juvenile salmon. Given this intention of enhanced outmigration benefits to juvenile threatened salmon, and the observed reductions in piscivory by predaceous fishes, the management practice of short-duration reservoir draining warrants further exploration.

An increased export of fishes, including invasive species, is hypothesized as one of the drivers leading to the differences in trophic levels observed here and could be of concern for downstream communities. However, invasive fishes are exported regularly from Willamette River reservoirs even during conventional management [14]. Although invasive fishes are regularly exported and are commonly found in the mainstem Willamette River below these study reservoirs, native fishes have still been documented as >90% of the fish in the mainstem Willamette River, likely due to habitat preferences [36].

The short duration (days) of the draining to riverbed maintains the ability of the reservoir to continue to meet multiple needs (e.g. flood risk reduction, summer recreation, and downstream flow augmentation), yet may have important consequences for the reservoir ecosystem through cascading trophic level impacts. It is unclear whether competition increases as trophic levels drop. We have not seen differences in zooplankton or benthic macroinvertebrate densities (S3 Table). If the driver of reduced piscivory is reduced densities of invertivorous prey fishes, the predators may be consuming invertebrates that would have been consumed by prey fishes under greater densities, rather than resulting in increased pressure on invertebrate prey.

Even in the era of high-profile removals, many large dams serve as part of critical flood control and power generating infrastructure and are likely to remain in place for some time. Thus, it is imperative to reduce their ecological impacts. It appears that unconventional short-duration draining of reservoirs simulates a pulse disturbance effective in temporarily improving downstream fish passage and ecosystem connectivity in addition to the lasting food web changes described here. However, additional studies are needed to fully understand the magnitude and mechanisms of these shifts in trophic positions across ecosystems. Reducing the negative ecological impacts of large dams through changes in their operations could present attractive management options with relatively modest costs.

## Materials and methods

### Study reservoirs

We compared food web dynamics in Fall Creek Reservoir (43°56′33″N 122°45′25″W), which was drained to riverbed for approximately 1 week each fall, to three nearby reservoirs with conventional reservoir management; Blue River (44°10′19″N 122°19′49″W), Hills Creek (43°40′16″N 122°25′33″W) and Lookout Point (43°53′48″N 122°43′34″W) Reservoirs, in the upper Willamette River basin, Oregon. The conventional management of the reference reservoirs includes dramatic seasonal changes in water levels and water storage (Fig 1), regularly exposing the benthic margins and likely limiting the littoral benthic community, both vegetation and benthic macroinvertebrates, due to repeated stranding and desiccation [37]. However, all three maintain some lake habitat (conservation pool) throughout the year. They also have comparable fish communities (S5 Table).

All of our study reservoirs are considered multipurpose structures that are kept at full pool during summer, for recreation and the capacity to maintain downstream flows, and have their water levels lowered starting in September to a minimum lake level, to provide flood control (Fig 1 and Fig 3). Fall Creek is not currently a power-generating facility, though it has been proposed [38]. The annual draining to riverbed of Fall Creek Reservoir began in 2011; two to three draining events had occurred prior to sampling in 2013 and 2014, respectively.

### Isotope sample collection and analyses

Reservoir biota were sampled during the productive summer months (June-August) of 2013 and 2014. Fish were captured in the central reservoir during full pool using large fyke net traps and short-set gill nets and lower caudal fin clips were collected (Oregon State University Institutional Animal Care and Use Permit 4476, Oregon Department of Fisheries and Wildlife permits 18168 and 18390, National Oceanic and Atmospheric Administration permits W1-13-OSU202 and W1-14-OSU202, and National Fish and Wildlife Service permit TE-31445B-D). Zooplankton were collected through the entire water column using a 1-m diameter, 64-μm mesh, open-tow net. Benthic invertebrates were collected using 1-m quadrats and kick nets in shallow waters along the reservoir margin and were sorted to family. Phytoplankton were collected from the same depth as peak chlorophyll-*a*, as determined by fluorescence profiles, and composited from five stations across each reservoir. Fishing efforts targeted a minimum sample size of five individuals per species per site, with more samples for more abundant species to allow for future mixing model analyses of diets.

Samples were retained on ice and returned to the lab for preparation for isotopic analyses of δ^13^C and δ^15^N. Samples were freeze-dried and sent to the Colorado Plateau Stable Isotope Laboratory (http://www.isotope.nau.edu/) for analyses for natural abundances of stable carbon and nitrogen isotope ratios (δ^13^C and δ^15^N). Ten percent of the isotope samples were randomly duplicated to ensure consistency.

### Statistical methods

We estimated a conservative threshold for piscivory in predaceous fishes (7.7‰) based on maximum mean δ^15^N trophic fractionation of 3.4 ‰ per trophic level [39], along with the observed δ^15^N of zooplankton to indicate basal resource δ^15^N (mean 0.88 ‰, n = 9, Table 1). Using this threshold, we identified fish of body length of >150 mm as likely being predominantly piscivorous in these reservoirs (Fig 2 C-D; r^2^ calculated using SigmaPlot 11 Dynamic Curve Fit. Largemouth Bass; y = 4.66 + (1.61*10–2)x–(6.19*10–6)x2; r2 = 0.82; Rainbow Trout; y = 4.66 + (1.61*10–2)x–(2.10*10–5)x2; r2 = 0.25)). We adopted the 150 mm size threshold based on consistent isotopic ratios of lower trophic levels that suggested similar fractionation rates across reservoirs. Thus, our analysis focused on inter-reservoir comparison of large predatory fishes rather than an exact quantification of piscivory per se. We assessed significance for differences in means using one-way ANOVA—Tukey multiple pairwise-comparisons (R Statistical Computing).

## Supporting information

S1 Table One-way ANOVA—Tukey multiple pairwise-comparisons for d15N signatures. (DOCX)

S2 Table Largemouth Bass Gut content sampling. (DOCX)

S3 Table Macroinvertebrate and Cladoceran densities in Fall Creek, Hills Creek and Lookout point reservoirs. (DOCX)

S4 Table Summer light extinction coefficients from study reservoirs. (DOCX)

S5 Table Fish community structure in study reservoirs. (DOCX)

S6 Table δ^15^N data for Largemouth Bass. (DOCX)

S7 Table δ^15^N data for Rainbow Trout. (DOCX)
