# Supplementary material for: Evidence for lasting alterations to aquatic food webs with short-duration reservoir draining
Source: PLoS One. 2019 Feb 7;14(2):e0211870. doi: 10.1371/journal.pone.0211870 (PMC6366690; doi:10.1371/journal.pone.0211870)
Supplement: S1 Table — (DOCX) [file pone.0211870.s001.docx]

**Table S1.** Results of one-way ANOVA - Tukey multiple pairwise-comparisons for δ15N values observed for large, greater than 150 mm fork length, Rainbow Trout (left) and Largemouth Bass (right) in treatment (Fall Creek) and reference (Blue River, Hills Creek, and Lookout Point) reservoirs. Statistically significant (p<0.05) results are bolded.

| Comparison | Rainbow Trout | | | | Largemouth Bass | | | |
| --- | --- | --- | --- | --- | --- | --- | --- | --- |
|  | Difference | Lower 95% CI | Upper 95% CI | p | Difference | Lower 95% CI | Upper 95% CI | p |
| **Blue River-Fall Creek** | **1.52** | **0.33** | **2.71** | **<0.01** | **1.99** | **0.22** | **3.76** | **0.02** |
| **Hills Creek-Fall Creek** | 1.06 | -0.28 | 2.41 | 0.17 | **2.34** | **1.04** | **3.63** | **<0.01** |
| **Lookout Point-Fall Creek** | **1.20** | **0.01** | **2.39** | **<0.05** | **2.16** | **0.87** | **3.45** | **<0.01** |
| Hills Creek-Blue River | -0.46 | -1.76 | 0.85 | 0.79 | 0.34 | -1.40 | 2.09 | 0.95 |
| Lookout Point-Blue River | -0.32 | -1.47 | 0.84 | 0.88 | 0.17 | -1.58 | 1.91 | 0.99 |
| Lookout Point-Hills Creek | 0.14 | -1.16 | 1.44 | 0.99 | -0.18 | -1.43 | 1.08 | 0.98 |
