# Supplementary material for: Evidence for lasting alterations to aquatic food webs with short-duration reservoir draining
Source: PLoS One. 2019 Feb 7;14(2):e0211870. doi: 10.1371/journal.pone.0211870 (PMC6366690; doi:10.1371/journal.pone.0211870)
Supplement: S2 Table — (DOCX) [file pone.0211870.s002.docx]

**Table S2.** Gut contents of Largemouth Bass collected opportunistically as mortalities from other sampling efforts (all sizes, few opportunistic mortalities were collected greater than 150 mm FL).

Note that δ15N indicates 150 mm as the size threshold where fish are predominantly piscivorous. We expect evacuation rates to be lower for ingested fish than ingested invertebrates and for %W (see below) values to be biased towards fish because they are larger prey.

|  | Fall Creek | Hills Creek | Lookout Point |
| --- | --- | --- | --- |
| Mean % fish (by dry weight) in stomach | 37% (n = 9) | 79% (n = 21) | 68% (n = 6) |
| Mean length of fish sampled (mm) | 138 | 82 | 105 |

Fish used were estimated to be dead less than 48 hrs before collection and freezing. Stomachs were removed and preserved in ethanol. Preserved stomachs were then examined under a dissecting scope, including enumeration and identification of contents by category (zooplankton, aquatic insect, terrestrial insect, terrestrial non-insect, fish and unknown). We report the percent contents by weight (%W), calculated using comparable field collected invertebrate (collected, sorted by family, and dried) and fish (measured, weighed and converted to dry weight assuming a wet to dry ratio of 5) samples.
