# Supplementary material for: Evidence for lasting alterations to aquatic food webs with short-duration reservoir draining
Source: PLoS One. 2019 Feb 7;14(2):e0211870. doi: 10.1371/journal.pone.0211870 (PMC6366690; doi:10.1371/journal.pone.0211870)
Supplement: S3 Table — (DOCX) [file pone.0211870.s003.docx]

**Table S3.** Average cladoceran zooplankton, total zooplankton, and benthic macroinvertebrate densities measured in the study reservoirs during June and August (full pool), 2014. The streambed draining treatment reservoir is highlighted in bold.

| Reservoir | Macroinvertebrate density  # / m^2^ | Cladocera density  # / m^3^ | Zooplankton density  # / m^3^ |
| --- | --- | --- | --- |
| Blue River Reservoir | not available | 171 | 510 |
| **Fall Creek Reservoir** | **29.6** | **376** | **985** |
| Hills Creek Reservoir | 30.3 | 167 | 533 |
| Lookout Point Reservoir | 29.4 | 289 | 1022 |

Zooplankton were collected through the entire water column using a 1-m diameter, 64-µm mesh, open-tow net and were identified to the lowest taxonomic resolution possible, typically genus or species. Benthic invertebrates were collected using 1-m quadrats and kick nets in shallow waters along the reservoir margin and were sorted to family.
