# Supplementary material for: Evidence for lasting alterations to aquatic food webs with short-duration reservoir draining
Source: PLoS One. 2019 Feb 7;14(2):e0211870. doi: 10.1371/journal.pone.0211870 (PMC6366690; doi:10.1371/journal.pone.0211870)
Supplement: S4 Table — (DOCX) [file pone.0211870.s004.docx]

**Table S4.** Light extinction coefficients calculated from light depth profiles collected using a Li-192 sensor in 2016.

| Month | Blue River | Fall Creek | Hills Creek | Lookout Point |
| --- | --- | --- | --- | --- |
| April | 0.343 | 0.761 | 0.522 | 0.572 |
| May | 0.337 | 0.435 | 0.468 | 0.468 |
| June | 0.330 | 0.441 | 0.388 | 0.388 |
| July | 0.300 | 0.424 | 0.349 | 0.349 |
| August | 0.299 | 0.396 | 0.390 | 0.390 |

Light as photosynthetic photon flux density was measured at 1-m intervals from surface to 30 m using a Li-192 sensor (LiCor, Lincoln, Nebraska). Light extinction coefficients represent the average value calculated from the log of light measured immediately below the water surface (L_0_) minus the log of the light at each depth (L_d_), divided by that depth (d).

k = (L_0_ - L_d_) / d

Extinction coefficient values were confirmed by fitting an exponential decay curve to measured values across depths, where the exponent represented the extinction coefficient.
