# Supplementary material for: Evidence for lasting alterations to aquatic food webs with short-duration reservoir draining
Source: PLoS One. 2019 Feb 7;14(2):e0211870. doi: 10.1371/journal.pone.0211870 (PMC6366690; doi:10.1371/journal.pone.0211870)
Supplement: S5 Table — (DOCX) [file pone.0211870.s005.docx]

**Table S5.** Top-four most common fish species found in downstream screw traps for each study reservoir from 2013-2015. Species listed for Blue River fishes are based on in reservoir sampling since no screw trap operated downstream.

| Blue River* | Fall Creek | Hills Creek | Lookout Point |
| --- | --- | --- | --- |
| Largemouth Bass | Bluegill | Chinook Salmon | Bluegill |
| Bluegill | Chinook Salmon | Sculpin | Chinook Salmon |
| Rainbow Trout | Largemouth Bass | Bluegill | Sculpin |
| Stickleback | Rainbow Trout | Rainbow Trout | White Crappie |
