# Supplementary material for: Evidence for lasting alterations to aquatic food webs with short-duration reservoir draining
Source: PLoS One. 2019 Feb 7;14(2):e0211870. doi: 10.1371/journal.pone.0211870 (PMC6366690; doi:10.1371/journal.pone.0211870)
Supplement: S6 Table — (DOCX) [file pone.0211870.s006.docx]

**Table S6.** Fork lengths (mm) and δ15N for Rainbow Trout. Grey boxes represent individuals greater than 150 mm FL. Considering all sizes, a one-way ANOVA (aov, R Statistical Computing), revealed a significant effect of size (p < 0.001) and treatment (p < 0.001; reference vs. Fall Creek; which experienced draining to streambed the prior fall) on δ^15^N. An ANCOVA (aov, R Statistical Computing) showed no significant interaction of size and treatment (P > 0.05).

| Fork Length (mm) | Blue River (δ15N) | Fork Length (mm) | Fall Creek (δ15N) | Fork Length (mm) | Hills Creek (δ15N) | Fork Length (mm) | Lookout Point (δ15N) |
| --- | --- | --- | --- | --- | --- | --- | --- |
| 116 | 4.39 | 127 | 5.12 | 80 | 5.78 | 75 | 6.62 |
| 142 | 5.37 | 231 | 4.81 | 83 | 7.47 | 123 | 6.11 |
| 219 | 9.83 | 255 | 6.95 | 88 | 4.95 | 140 | 8.09 |
| 230 | 7.46 | 260 | 5.23 | 91 | 4.91 | 160 | 6.43 |
| 237 | 9.79 | 261 | 6.09 | 111 | 6.31 | 245 | 6.34 |
| 245 | 6.63 | 284 | 7.00 | 120 | 8.02 | 247 | 8.91 |
| 249 | 6.41 | 310 | 6.74 | 128 | 6.32 | 271 | 7.30 |
| 251 | 6.79 | 315 | 6.73 | 132 | 7.23 | 302 | 8.32 |
| 268 | 6.23 | 327 | 6.41 | 135 | 8.16 | 305 | 6.21 |
| 277 | 7.49 | 331 | 9.25 | 152 | 8.44 | 320 | 6.99 |
| 279 | 8.61 | 338 | 5.87 | 161 | 6.19 | 333 | 7.49 |
| 285 | 8.94 | 409 | 4.75 | 175 | 5.65 | 343 | 7.71 |
| 292 | 6.97 | 462 | 6.49 | 181 | 7.34 | 359 | 7.95 |
| 305 | 8.11 |  |  | 219 | 6.81 | 362 | 9.14 |
| 334 | 8.48 |  |  | 230 | 6.72 | 364 | 6.32 |
| 344 | 8.57 |  |  | 248 | 8.34 | 378 | 8.06 |
|  |  |  |  | 270 | 8.16 | 380 | 8.69 |
|  |  |  |  | 270 | 9.15 |  |  |
