# Supplementary material for: Evidence for lasting alterations to aquatic food webs with short-duration reservoir draining
Source: PLoS One. 2019 Feb 7;14(2):e0211870. doi: 10.1371/journal.pone.0211870 (PMC6366690; doi:10.1371/journal.pone.0211870)
Supplement: S7 Table — (DOCX) [file pone.0211870.s007.docx]

**Table S7.** Fork lengths (mm) and δ15N for Largemouth Bass. Grey entries represent individuals greater than 150 mm FL. Considering all sizes, a one-way ANOVA (aov, R Statistical Computing), revealed a significant effect of size (P < 0.001) and treatment (P < 0.001; reference vs. Fall Creek; which experienced draining to streambed the prior fall) on δ15N. An ANCOVA (aov, R Statistical Computing) showed no significant interaction of size and treatment (P > 0.05).

| Fork Length (mm) | Blue River (δ15N) | Fork Length (mm) | Fall Creek (δ15N) | Fork Length (mm) | Hills Creek (δ15N) | Fork Length (mm) | Lookout Point (δ15N) |
| --- | --- | --- | --- | --- | --- | --- | --- |
| 60 | 3.88 | 62 | 6.47 | 39 | 6.04 | 91 | 5.78 |
| 75 | 5.78 | 96 | 4.75 | 53 | 6.53 | 95 | 5.83 |
| 81 | 6.82 | 106 | 5.50 | 66 | 5.09 | 101 | 6.31 |
| 95 | 4.38 | 107 | 5.19 | 72 | 5.11 | 114 | 7.02 |
| 99 | 5.60 | 114 | 4.69 | 85 | 6.98 | 129 | 8.88 |
| 151 | 7.13 | 130 | 5.32 | 92 | 5.32 | 142 | 6.28 |
| 225 | 7.89 | 132 | 5.35 | 100 | 5.65 | 152 | 8.11 |
| 228 | 7.64 | 134 | 4.76 | 116 | 5.82 | 160 | 7.84 |
| 244 | 7.31 | 137 | 4.78 | 134 | 7.16 | 162 | 7.70 |
| 389 | 8.76 | 138 | 4.48 | 141 | 7.56 | 173 | 6.89 |
| 438 | 9.48 | 139 | 4.82 | 146 | 6.50 | 218 | 8.01 |
| 494 | 11.98 | 140 | 5.76 | 165 | 7.89 | 240 | 7.69 |
|  |  | 141 | 4.97 | 170 | 7.13 | 247 | 9.95 |
|  |  | 142 | 5.85 | 184 | 6.65 | 259 | 8.10 |
|  |  | 145 | 5.10 | 195 | 6.95 | 271 | 8.09 |
|  |  | 151 | 4.76 | 199 | 7.21 | 288 | 9.10 |
|  |  | 152 | 5.27 | 200 | 6.51 | 292 | 9.43 |
|  |  | 154 | 8.24 | 201 | 6.98 | 295 | 8.87 |
|  |  | 155 | 5.62 | 209 | 6.95 | 301 | 9.63 |
|  |  | 156 | 5.28 | 225 | 8.79 | 304 | 9.23 |
|  |  | 158 | 5.69 | 236 | 8.53 | 307 | 9.18 |
|  |  | 160 | 5.67 | 276 | 12.84 | 315 | 9.74 |
|  |  | 165 | 6.03 | 300 | 9.68 | 352 | 9.66 |
|  |  | 167 | 5.92 | 303 | 8.11 | 359 | 9.37 |
|  |  | 169 | 8.96 | 347 | 10.50 | 390 | 9.15 |
|  |  | 170 | 7.56 | 353 | 10.16 | 407 | 9.56 |
|  |  | 171 | 6.24 | 418 | 10.41 |  |  |
|  |  | 177 | 5.00 | 419 | 9.62 |  |  |
|  |  | 178 | 6.28 | 451 | 11.31 |  |  |
|  |  | 183 | 6.48 | 459 | 11.05 |  |  |
|  |  | 195 | 7.79 | 538 | 11.55 |  |  |
|  |  | 338 | 9.19 |  |  |  |  |
|  |  | 352 | 8.91 |  |  |  |  |
